# Multidisciplinary Treatment of Liver Metastases from Intracranial SFTs/HPCs: A Report of Three Consecutive Cases

**DOI:** 10.3390/curroncol29110687

**Published:** 2022-11-15

**Authors:** Felix J. Krendl, Franka Messner, Gregor Laimer, Angela Djanani, Andreas Seeber, Georg Oberhuber, Dietmar Öfner, Dominik Wolf, Stefan Schneeberger, Reto Bale, Christian Margreiter

**Affiliations:** 1 Department of Visceral, Transplant and Thoracic Surgery, Center for Operative Medicine, Medical University of Innsbruck, 6020 Innsbruck, Austria; 2 Department of Radiology, Section of Interventional Oncology—Microinvasive Therapy, Medical University of Innsbruck, 6020 Innsbruck, Austria; 3 Clinical Division of Gastroenterology, Hepatology and Metabolism, Department of Internal Medicine, Medical University of Innsbruck, 6020 Innsbruck, Austria; 4 Department of Hematology and Oncology, Medical University of Innsbruck, 6020 Innsbruck, Austria; 5 Pathology Department, Medical University of Innsbruck, 6020 Innsbruck, Austria

**Keywords:** solitary fibrous tumor, hemangiopericytoma, SFT/HPC, liver metastases, SRFA, multidisciplinary treatment

## Abstract

In the 2016 WHO classification of tumors of the central nervous system, hemangiopericytomas (HPCs) and solitary fibrous tumors (SFTs) were integrated into a new entity (SFT/HPC). Metastases to bone, liver, lung, and abdominal cavity are of concern. Only 37 cases of patients with liver metastases due to intracranial SFTs/HPCs have been reported. Herein, we present our experience in the management of patients with liver metastases from intracranial SFTs/HCPs. All consecutive patients who were treated for liver metastases from intracranial SFTs/HPCs from January 2014 to December 2020 were enrolled. Overall, three patients were treated for liver metastasis from SFTs/HPCs with curative intent. Two patients with bilobar metastases at presentation required surgical resection, transarterial embolization, stereotactic radiofrequency ablation (SRFA) and systemic therapy. One patient with a singular right liver lobe metastasis was treated with SRFA alone. This patient shows no evidence of liver metastases 39 months following diagnosis. Of the two patients with bilobar disease, one died 89 months following diagnosis, while one is still alive 73 months following diagnosis. Long-term survival can be achieved using a multimodal treatment concept, including surgery, loco-regional and systemic therapies. Referral to a specialized tertiary cancer center and comprehensive long-term follow-up examinations are essential.

## 1. Introduction

Solitary fibrous tumors (SFTs) and hemangiopericytomas (HPCs) are rare mesenchymal tumors of fibroblastic type that may occur at various locations across the body including the central nervous system [1]. Historically, HPCs have been thought to derive from Zimmerman’s pericytes, which are modified smooth muscle cells lining capillaries, postcapillary venules and sinusoidal spaces. However, the HPC-like vascular pattern was also seen in other soft tissue sarcomas including SFT [2,3]. Therefore, whether HPCs constitute a distinct pathologic entity, or a non-specific vascular pattern has long been a matter of debate among pathologists [2]. HPCs and SFTs have many clinical and morphological features in common, yet they differ in terms of cellular and stromal composition and even in terms of expressed cell surface markers such as CD34 and CD99, which has previously led to confusion and diagnostic uncertainty [3].

This is why, up until recently, SFTs and HPCs have been considered as different histomorphological clinical entities [4,5]. Recent work, however, has shown that these tumors display widely overlapping molecular features making them rather identical entities with varying biological behavior [3,6,7]. Both, SFTs and HPCs, share genomic inversions at the 12q13 locus, fusing the NAB2 and STAT6 genes, which leads to abnormal STAT6 gene expression [8,9,10,11]. Therefore, the presence of abnormal STAT6 gene expression has become the diagnostic hallmark of SFTs/HPCs and HPC is now considered a more cellular variant of SFTs [12].

Consequently, in the 2016 WHO classification of tumors of the central nervous system (CNS), HPCs and SFTs were summarized into a new entity (SFT/HPC) [7]. Unlike other intracranial tumors, where each entity is usually assigned a specific grade, SFTs/HPCs are assigned three grades based on their histopathologic appearance (Table 1). Their malignant potential varies significantly based on the primary tumor grading, with metastatic disease possibly occurring years after the initial diagnosis [1,3]. The extracranial metastatic rate for SFTs/HPCs has been reported to be as high as 68%, with bone, liver, lung, and the abdominal cavity being the most common sites of metastasis [1,13,14,15,16].

Reports of patients with liver metastases due to intracranial SFTs/HPCs are rare [17,18]. Therefore, no standard of care has been established. Due to rapid advances in the medical field over the past decades, today, a large armamentarium including loco-regional, surgical, and systemic treatment options is available. Choosing the right treatment at the right time for the right patient is challenging. Herein, we present our experience in the management of patients with liver metastases from intracranial SFTs/HCPs.

## 2. Materials and Methods

For this case series all consecutive adult patients who were treated for liver metastases from intracranial SFTs/HPCs at our tertiary academic cancer center from January 2014 to December 2020 were enrolled (*n* = 3). Patient data was collected as part of a prospectively maintained database. All patients consented to have their clinical data recorded in our database. The prospective data collection was approved by the local ethics committee (Nr.: 300/4.17). The outcome analysis was performed retrospectively. This case series has been reported in line with the PROCESS criteria [19]. This research study was registered in a publicly accessible database number in accordance with the Declaration of Helsinki (Research Registration ID: researchregistry7744).

In patients with paraneoplastic syndromes, hypercalcemia and hypoglycemia were corrected before any treatment interventions. Hypercalcemia was treated with hydration and zoledronic acid while hypoglycemia was corrected with intravenous glucose infusion and high-caloric nutrition. All patients were discussed in a multidisciplinary hepatobiliary tumor board at the time of diagnosis as well as following completion of the recommended treatments or when progressive disease was found upon follow-up. Patients were treated with a combination of loco-regional therapies, such as stereotactic radiofrequency ablation (SRFA), or transarterial embolization (TAE), as well as surgical resection and systemic therapies. Percutaneous and trans-arterial loco-regional therapies were considered as first-line treatment options in all cases due to their parenchyma sparing nature and the high likelihood of distant tumor recurrence. In addition, in two cases, initial surgical resection with curative intent would not have been possible due to the bilobar distribution of the metastases. Surgical resection was considered if tumor control with loco-regional modalities was not successful. Systemic therapy was only considered in patients in whom local control could not be achieved with loco-regional therapies and surgery. SRFA and TAE were performed as described below.

Briefly, the SRFA procedure [20,21] was performed in a dedicated intervention room with a sliding gantry CT (SOMATOM Sensation open, Siemens Inc., Munich, Germany), moving on rails between two different rooms. All procedures were performed under general anesthesia with full muscle relaxation. Respiratory triggering (mandatory for exact registration) was achieved by temporary endotracheal tube disconnection. Patients were immobilized using a single (Bluebag, Medical Intelligence, Schwabmünchen, Germany) or double vacuum fixation technique (BodyFix, Medical Intelligence, Schwabmünchen, Germany). After acquiring a dual-phase contrast enhanced planning CT scan (arterial/portal venous phase; 3 mm slice thickness), all data was transferred to the optical based 3D navigation system (S8, Medtronic Inc., Dublin, Ireland). Using the navigation systems software multiple needle trajectories were planned on multi-planar and 3D reconstructed images. Subsequently, 15G/17.2 cm coaxial needles (Bard Inc., Covington, USA) were inserted through the ATLAS aiming device (Interventional Systems Inc., Kitzbühel, Austria), allowing a navigated alignment of the planned trajectories without real-time imaging. Correct needle placement was verified with a non-enhanced CT scan, superimposed onto the planning CT scan, with the possibility of manual re-adjustment. After that, up to three 17G RF-electrodes at a time (25 cm length; 3 cm exposure) were introduced through the coaxial needles and thermal ablation began using a unipolar RF generator (Cool-tip, Medtronic Inc., Dublin, Ireland). Needle track cauterization was performed during every repositioning and at final probe removal to prevent bleeding and potential tumor seeding. Finally, a dual-phase control CT scan (arterial/portal venous phase; 3 mm slice thickness) was obtained to detect possible complications (i.e., bleeding, pneumothorax) and to verify complete tumor ablation including a sufficient safety margin. In case of incomplete ablation (i.e., residual tumor; lack of safety margin), stereotactic placement of additional coaxial needles with subsequent serial ablation may be performed.

For TAE [22], the feeding arteries to the lesion or lesions were catheterized as selectively as possible and occluded under fluoroscopic control using different materials. All procedures were performed in an angiographic suite (Integris BV 3000, Philips; Multistar, Siemens, Munich, Germany) under local anesthesia. Following diagnostic catheter angiography using Lipiodol^®^ (Guerbet LCC, Villepinte, France) the feeding arteries to the lesion or lesions were catheterized as selectively as possible using a Cantata^®^ microcatheter (Cook Medical, Bloomington, IN, USA) and a Fathom™ 16 steerable guidewire (Boston Scientific, Marlborough, MA, USA). Embolization of the feeding vessels was achieved applying one or a combination of the following materials: Embozene™ Microspheres (CeloNova BioSciences, Inc., San Antonio, TX, USA), IDC™ Soft Embolization Coils (Boston Scientific, Marlborough, MA, USA), Pushable Fibered and Detachable Non-Fibered Microcoils (Cook Medical, Bloomington, USA), MicroPlex^®^ Coil System (MicroVention, Inc., Aliso Viejo, CA, USA) and Contour™ PVA Embolization Particles (150–500 μm, Boston Scientific, Marlborough, MA, USA). In all patients, a completion angiography using Lipiodol^®^ (Guerbet LCC, Villepinte, France) was performed to document effective embolization of the feeding vessels and to rule out embolization of non-target vessels. Both, TAE and SRFA, were exclusively performed by experienced interventional radiologists.

Surgical resections were performed following standard surgical techniques. All procedures were performed under general anesthesia with the patient placed in a supine position. Following laparotomy, the hilar structures were dissected, identified, and transected. The parenchyma was then transected using the cavitron ultrasonic surgical aspirator (CUSA), bipolar forceps and clips. Liver veins were either stapled or clamped and oversewn following transection. A surgical drain was routinely placed during all liver resections. All resections were performed by surgeons with extensive experience in HPB and liver transplant surgery.

For systemic therapy, patients received either axitinib 5 mg per os (PO) twice a day (BID) or pazopanib 400 mg PO BID until progression or limiting toxicity. Dosing of temozolomide was based on the patient’s body surface area (150 mg/m^2^) and administered orally on days 1–7 and days 15–21 while bevacizumab (5 mg/kg) was given intravenously on day 8 and day 22 on a 28-day cycle.

## 3. Results

Three female patients with a prior history of intracranial SFT/HPC aged between 39 and 67 years at the time of their liver metastases diagnosis, were enrolled in this study. All three patients had been diagnosed with an intracranial SFT/HPC before developing liver metastases. In all patients, the intracranial SFT/HPC was resected with curative intent at the time of the initial diagnosis. All patients received adjuvant radiotherapy.

**Figure 1:**
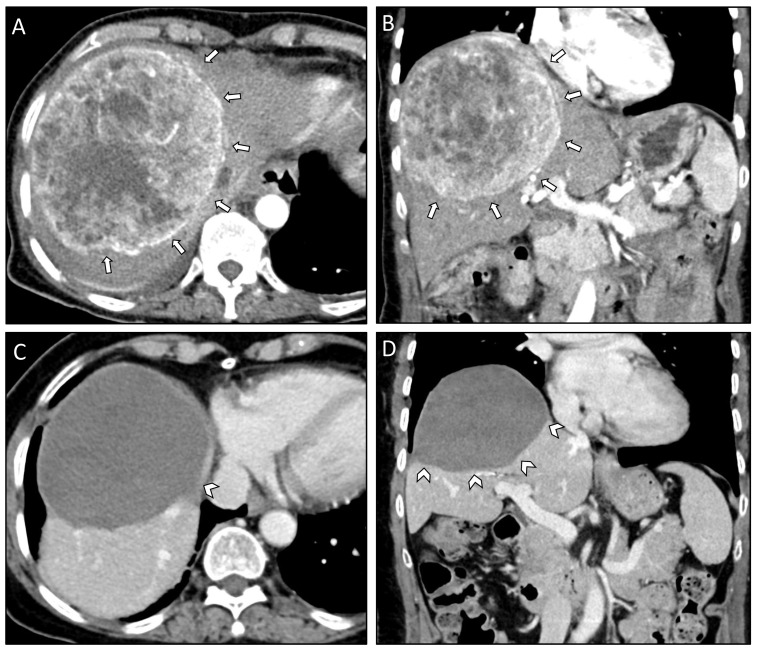
Axial (**A**) and coronal plane (**B**) of the pre-interventional CT scan of case #1 depicting a 13 cm measuring SFT/HPC in the right liver lobe (white arrows). Corresponding axial (**C**) and coronal plane (**D**) of the last follow-up CT scan 36 months after the initial diagnosis of liver metastasis and 11 months after the last SRFA session with progressively shrinking coagulation zone (white arrowheads).

Time from the initial SFT/HPC diagnosis to the occurrence of liver metastases ranged between eight and 15 years. Grading of the SFT/HPC, according to the WHO classification, showed grade II tumors in all three patients. Two out of three patients presented with paraneoplastic syndromes (hypercalcemia and hypoglycemia). Two patients presented with bilobar liver metastases while one patient suffered from a singular right lobe metastasis. The size of the largest metastasis was similar in all patients and ranged from 12 to 13 cm in diameter (Figure 1). In all three cases, the liver lesions were biopsied for histopathologic workup, which confirmed metastatic disease from an SFT/HPC. For the most recently diagnosed case (case #1), STAT6 immunohistochemistry (IHC) testing results were available, indicating the presence of abnormal STAT6 expression in the biopsied lesion. In the other two cases, IHC staining was positive for CD99 with case #2 additionally staining positive for CD34 and case #3 showing expression of Vimentin (Table 2).

Ιn case #1, a total of six SRFA sessions (Table 3) were required to achieve complete local tumor control in liver segments V, VII and VIII (Figure 1A,B). Five months later, a metastasis in the omentum majus was detected and laparoscopically resected. Histopathologic examination of the excised tumor showed in-toto resection (R0) and STAT6 positive tumor cells with more than 10 mitoses per HPF and a Ki-67 index of 15–20% indicating recurrence of a higher grade SFT/HPC (WHO grade III). The postoperative course was uneventful, and the patient was discharged on postoperative day (POD) six. At the last follow-up, the patient’s liver was tumor free (Figure 1C,D), eleven years following the initial SFP/HPC diagnosis and 39 months following the diagnosis of liver metastases. However, multiple osteolytic lesions suspicious for bone metastases were seen on the last imaging studies and the patient was started on denosumab (Table 4).

**Figure 2:**
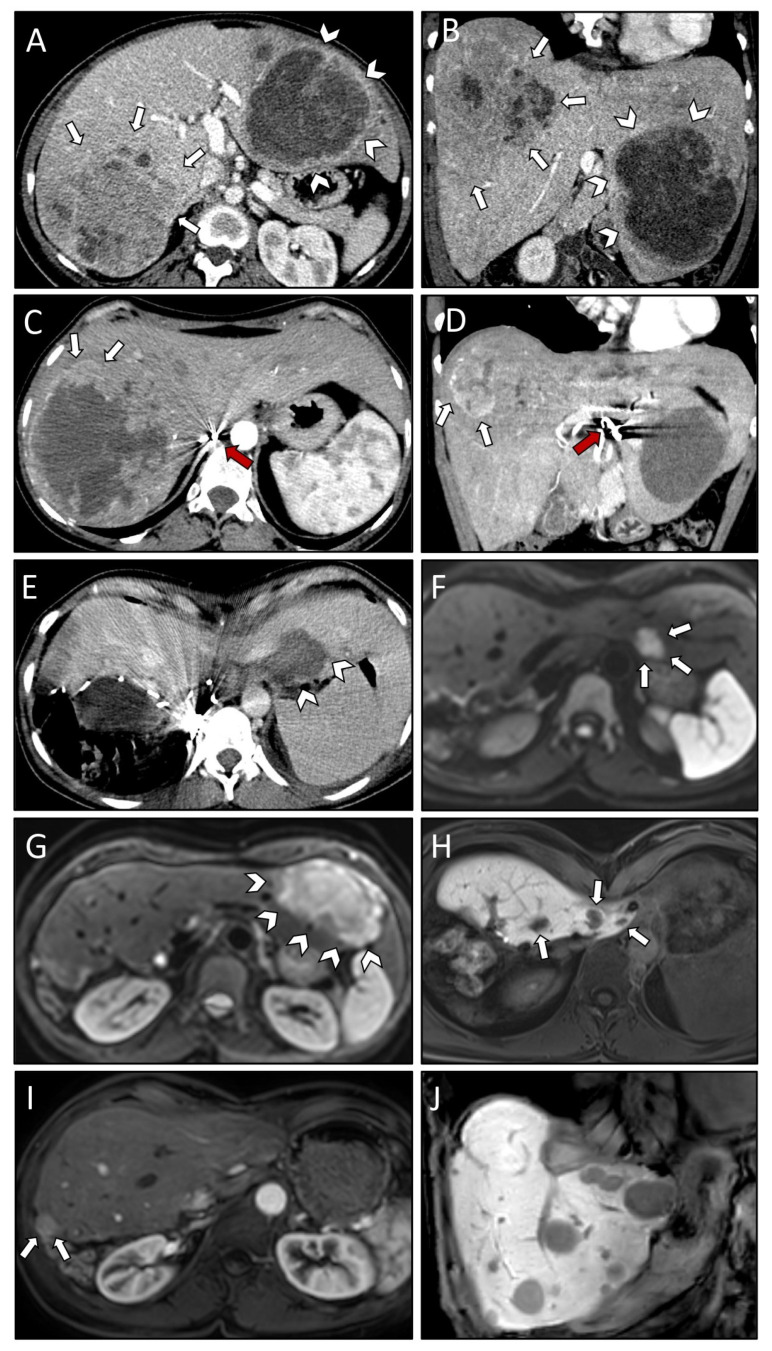
Axial (**A**) and coronal plane (**B**) of initial CT scans demonstrating very large SFT/HPC liver metastases in both right (white arrows) and left (white arrowheads) liver lobe. CT scan in axial (**C**) and coronal plane (**D**) after three TAE sessions revealing insufficient devascularization of the right liver lobe lesion anteriorly (white arrows). Note the beam hardening artifacts (red arrows) in the hepatic artery. Post-interventional CT scan (**E**) after first SRFA with laparoscopic liver packing (protection of cardio-esophageal region) showing coagulation zone (white arrowheads) with no residual vital tumor tissue visible. Contrast-enhanced MRI scan four months after first SRFA revealing a new metastasis in liver segment II (white arrows; (**F**)) and local recurrence in segment III following TAE (white arrowheads; (**G**)). MRI scan depicting three of six new liver lesions in segment IVa (white arrows; (**H**)) after resection of left lateral segments, all of them successfully treated with SRFA. MRI scans 15 months after the last intervention showing local recurrence at the resection margin (white arrows; (**I**)) and multiple new liver metastases (**J**).

Case #2 underwent three TAE sessions for the large lesion in the right and left liver lobe (Figure 2A,B). An infection of necrotic liver tissue was observed as a post-interventional complication following two of the TAE treatment sessions and the patient received antibiotic therapy (Clavien–Dindo [CD] II) (Table 3). Due to insufficient devascularization (Figure 2C,D), the tumor in the right liver lobe was finally removed via a right hemihepatectomy. The postoperative course was uneventful, and the patient was discharged on POD eight. Two months later, SRFA with simultaneous laparoscopic liver packing [23] was scheduled to treat the remaining liver metastasis in segment II. Laparoscopic liver packing was indicated to protect the cardio-esophageal region during the SRFA treatment session. CT scans immediately following the SRFA treatment did not show any residual tumor (Figure 2E). Four months later, a new metastasis in segment II and a local recurrence in segment III (following TAE) was observed (Figure 2F,G). Therefore, the left lateral segments were resected. The patient recovered without any complications and was discharged on POD six.

Follow-up CT scans revealed six new liver lesions in segment IVa (Figure 2H), which were treated in three subsequent SRFA treatment sessions. During 3-month follow-up imaging intervals the patient remained in full remission for 15 months until multiple new liver metastases and local recurrence at the resection margin were found on follow-up scans (Figure 2I). This time, rescue liver transplantation was discussed with the patient as a last resort and systemic therapy with the multikinase inhibitor axitinib was initiated as a bridge to transplantation. However, the patient opted against liver transplantation and hence systemic therapy was continued. Three months later, the patient developed intracranial recurrence, which was surgically resected. The histopathologic report showed recurrence of the known SFT/HPCs although this time the tumor recurrence was classified as WHO grade III. Four months later, the follow-up MRI showed a residual brain tumor. Adjuvant radiotherapy was recommended. At the same time, both MRI as well as PET-CT scans showed multiple new liver lesions compatible with SFT/HPCs metastases indicating progressive disease. One of the new liver lesions was biopsied to obtain tumor tissue for whole exome and whole transcriptome sequencing (at Caris Life Sciences). The molecular analysis showed a NAB2-STAT6 gene fusion, again confirming the SFT/HPCs diagnosis. However, no targetable mutations were found and systemic therapy with temozolomide and bevacizumab was recommended. At the last follow-up, following seven cycles of systemic therapy, multiple lesions in the remaining liver parenchyma were observed (Figure 2J) indicating progressive disease. The patient remains AWD 73 months following the diagnosis of liver metastases and 14 years after the initial diagnosis of the primary tumor (Table 4).

**Figure 3:**
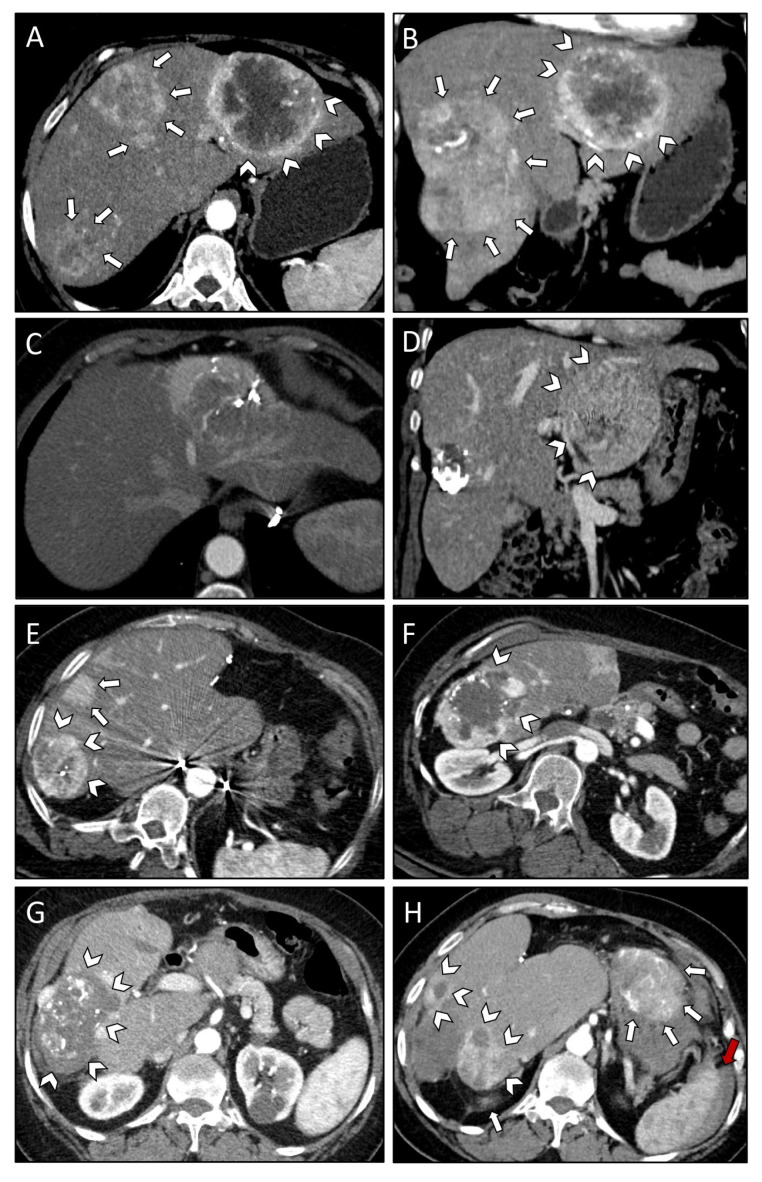
Axial (**A**) and coronal plane (**B**) of initial CT scans demonstrating multiple large SFT/HPC liver metastases affecting both right (white arrows) and left (white arrowheads) liver lobe. Axial (**C**) and coronal (**D**) plane of control CT scans following a total of six TAE sessions revealing mixed response with residual vital tumor tissue in liver segment II/III (white arrowheads; (**D**)). CT scans nine months after resection of left lateral segments revealing recurrent lesions in segments V, VI, VII (white arrowheads; (**E**,**F**)) and two new lesions in segments IVa and VII (white arrows; (**E**)). Successful treatment of recurrent lesions after a total of six SRFA and one additional TAE session as shown in the first follow-up CT scan after the last SRFA (white arrowheads; (**G**)). CT scan obtained following syncope 18 months after the last intervention revealing diffuse metastatic liver disease (white arrowheads; (**H**)), peritoneal metastases (white arrows; (**H**)), and ascites (red arrow; (**H**)) 18 months after the last intervention.

For case #3, six TAE treatment sessions were necessary to deal with lesions in segments II, III, IVa and IVb, as well as segments V, VI and VII (Figure 3A,B). The patient developed post-embolization syndrome (CD I) but was otherwise fine (Table 3). Re-staging CT scans showed a mixed response, with metastases remaining in segments II and III (Figure 3C,D). Surgical resection of the left lateral segments was recommended by the local multidisciplinary hepatobiliary tumor board and subsequently performed. Postoperatively, the patient developed a bilioma, which was treated by drainage via the intraoperatively placed surgical drain (CD I). The patient was discharged on POD 22. Nine months after surgery, recurrent lesions in segments V, VI, VII and two new lesions in segments IVa and VII were observed (Figure 3E,F). These lesions were successfully treated through the combination of one TAE and five SRFA treatment sessions. The patient developed a post-interventional right-sided pleural effusion following one of the SRFA treatment sessions, which was treated with a pleural pigtail-catheter (CD IIIa). Due to the development of new liver lesions in segments V and VIII and local recurrence in segments V, VI and VII three months later, another SRFA session had to be performed. The follow-up CT scans showed successful treatment of all recurrent lesions (Figure 3G). Eight months later, the patient presented with left lower extremity pain, which was related to osteolytic lesions in the left femur and acetabulum being highly suspicious of SFT/HCP metastases. Biopsy of the osteolytic lesions was inconclusive. The highly vascularized metastases were embolized (TAE) and the left femur was stabilized with an intramedullary nail. Palliative radiotherapy of the acetabulum and systemic off-label therapy with pazopanib was initiated. While under systemic therapy with pazopanib, the patient developed sepsis with acute kidney injury (NCI Common Terminology Criteria for Adverse Events, [CTCAE] grade III). Systemic therapy was stopped, and antibiotic therapy was started. The patient recovered from her sepsis and was discharged. The patient did not appear at her scheduled follow-up appointments and presented to the emergency department with acute abdominal pain and syncope 18 months later. CT scans revealed diffuse metastatic liver disease and peritoneal metastases with ascites (Figure 3H). In addition, progression of the osseous metastases was observed. The patient received hospice care and died 22 years following the initial SFT/HPC diagnosis and 89 months following the diagnosis of metastatic disease to the liver (Table 4).

## 4. Discussion

Metastatic disease to the liver is relatively common in patients with intracranial SFTs/HPCs and may occur early or late following the initial diagnosis [1]. Once metastatic spread has occurred, curative treatment seems out of reach. However, our case study shows that, choosing from a variety of treatment options, including loco-regional modalities such as TAE and SRFA as well as surgical resection and systemic therapies, long-term survival may be possible. Yet, the net survival benefit achieved with aggressive treatment compared to a more conservative approach remains unclear. Most of the available evidence comes from case reports and case series without appropriate controls. Furthermore, the previously inconsistent reporting in terms of tumor grading, the recent WHO reclassification of SFTs/HPCs as well as patient heterogeneity make comparison of individual cases difficult. In an analysis of 38 patients with HPCs (SFTs/HPCs WHO grade II and III) of the CNS treated at the Mayo Clinic metastatic disease, not local recurrence, was found to be the most common cause of death, indicating the importance of systemic tumor control [24]. Similarly, a recent systematic review found that failure to control metastatic disease in patients with SFTs/HPCs results in a significant reduction in survival [25].

Consequently, these patients should be managed at specialized academic cancer centers where such treatment options can be offered. A multidisciplinary treatment approach tailored towards the patient’s individual needs and systemic disease burden is crucial.

The clinical presentation of metastatic disease from SFTs/HPCs is often vague and indolent. However, paraneoplastic syndromes have been described [26,27,28,29,30]. In this case, series two of three patients suffered from paraneoplastic syndromes at the time of the hepatic metastases diagnosis. One patient presented with hypoglycemia, while the other suffered from hypercalcemia. Hypoglycemia has previously been described in the context of SFT/HPCs and, is commonly referred to as Doege–Potter syndrome in this setting [31,32,33,34,35,36,37,38,39,40]. Insulin-like growth factors (IGFs), produced by the tumor, have been implicated in the pathogenesis of Doege–Potter syndrome [31,32]. Hypoglycemia can be treated with intravenous glucose infusion and resection of the hormone producing tumor is curative [32,34]. Hypoglycemic episodes may act as a warning sign when following patients with SFTs/HPCs since hypoglycemia more likely occurs in patients with extracranial metastatic disease [29].

Paraneoplastic hypercalcemia is a common finding affecting up to 30% of patients with malignancies [39]. Hypercalcemia may be due to the secretion of parathyroid hormone-related protein, osteolytic activity at the site of osseous metastases or secretion of vitamin D or ectopic parathyroid hormone from the tumor itself [40]. Treatment consists of aggressive intravenous (IV) hydration and IV bisphosphonates, such as pamidronate and zoledronic acid [41].

The reported time frame from initial diagnosis of intracranial SFTs/HPCs to the occurrence of metastatic disease ranges from three months to 31 years, with the mean time to metastasis reported at 8 years [1,16,42]. Recently, a case of synchronous metastatic disease to the liver has been reported in a patient with a highly aggressive WHO grade III intracranial SFT/HPC (Table 5) [17]. In our cohort, all patients had WHO grade II SFTs/HPCs and developed metastatic disease between eight and 15 years following the initial SFT/HPCs diagnosis. Although the overall survival is lower in patients with more aggressive WHO grade III tumors, metastatic disease appears to be more common in patients with WHO grade II tumors [43]. Long-term periodic follow-up with full body cross-sectional imaging studies is mandatory.

In addition to imaging studies, biopsy and histopathologic workup of suspicious lesions is required. Along with standard histology, IHC is mandatory to establish the diagnosis. Previously, CD34, CD99 and Vimentin were the preferred diagnostic IHC markers; however, varying expression has sometimes led to diagnostic uncertainty. In a study by Bouvier et al. [3] that analyzed histological and immunohistochemical features of SFTs/HPCs, all tumors stained positive for Vimentin regardless of tumor grade. Expression of CD99 was found to be more likely in higher grade tumors (WHO grade II and III) with CD34 expression more commonly observed in low grade tumors (WHO grade I). More recently, with the discovery of the pathognomonic NAB2-STAT6 gene fusion through whole-genome sequencing, STAT6 has moved into the diagnostic spotlight. NAB2-STAT6 gene fusion leads to nuclear translocation of STAT6, which can then be detected by IHC with high sensitivity and specificity [12]. In our series, STAT6 IHC testing results were available for the most recently diagnosed case (case #1), confirming the presence of abnormal STAT6 expression in the biopsied lesion. In the other two cases, IHC staining was positive for CD99 with case #2 additionally staining positive for CD34 and case #3 showing expression of Vimentin (Table 2).

Once the diagnosis of hepatic metastasis from SFT/HPC has been established, a wide variety of treatment options are available. Historically, surgical resection has been the mainstay of treatment [18,45,51]. In two of our reported cases (#2 and #3), surgical resection was necessary to achieve local tumor control. Reports of metastasectomies date back to the early 1960s [45], and surgical resection remains an important cornerstone in the management of liver metastases to this day (Table 5) [18,60,63,66].

Recently, loco-regional treatment options and systemic therapies were introduced as part of a multidisciplinary treatment approach and enabled good clinical outcomes. Transcatheter arterial embolization (TAE) was first reported as a treatment option in 1996 [31]. Since then, both TACE and TAE have been successfully applied in multiple cases (Table 5) [38,58,63,64]. Radiofrequency ablation (RFA) is another promising loco-regional treatment modality that has been used to treat liver metastases in the context of SFTs/HPCs [58,60,63,66].

Using a combination of TACE and RFA, Iwamuro et al. were able to achieve local tumor control without evidence of hepatic recurrence at a five-year follow-up [58]. Lo et al. [66] reported two cases in which RFA was used to treat liver metastases from SFTs/HPCs. In one case, RFA was combined with surgical resection and the patient was alive with disease (AWD) 36 months following diagnosis but later lost to follow-up. In the second case, RFA was used without combining other treatment options and the patient died of disease (DOD) 36 months following diagnosis.

Adding to the previously described loco-regional treatment options, Reddy et al. [17] recently published a case where stereotactic body radiation therapy (SBRT) was used to treat liver metastases from an intracranial SFT/HPC. Two liver lesions in segments VI and VIII were treated with 40 Gy and 60 Gy in one and five fractions, respectively. An MRI of the abdomen obtained six weeks following the completion of radiotherapy showed disease progression of the hepatic metastases and systemic therapy was initiated. Radiotherapy has been used successfully to treat liver metastases of various origins [69,70], yet in the context of SFTs/HPCs, the literature on SBRT is scarce. For now, SBRT can be considered part of the available loco-regional treatment options, however its role within the context of SFTs/HPCs remains to be defined.

While we have not used TACE or SBRT in our case series, we used a combination of TAE and SRFA as part of our multidisciplinary treatment strategy with good clinical outcomes (Table 3).

Multiple studies investigated the effect of systemic therapies in patients with SFTs/HPCs. Anthracycline-based chemotherapies (i.e., doxorubicin) have been used as front-line therapies in the treatment of SFTs/HPCs. However, overall response rates appear to be low and data regarding their efficacy is limited [60,71]. More recently, the combination of temozolomide, the oral pro-drug of dacarbazine, an alkylating agent, and bevacizumab, a monoclonal antibody targeting the vascular endothelial growth factor (VEGF) was reported to be effective in patients with SFT/HPCs [72]. As SFTs/HPCs are highly vascularized tumors, exploring antiangiogenic treatment options seems to be worthwhile and good outcomes have been observed [73].

A retrospective analysis by Park et al. [72] at the MD Anderson Cancer center, including 14 patients with histologically confirmed SFTs/HPCs who were treated with temozolomide and bevacizumab, showed partial response in 11 patients (79%) according to Choi criteria. Stable disease was seen in two patients (14%) while one patient (7%) had disease progression.

Reddy et al. [17] reported a patient with SFT/HPC metastases to the liver, with stable disease when seen at the last follow-up 18 months following diagnosis, after the use of a combination of SBRT for local tumor control and systemic antiangiogenic therapy with temolozomide and bevacizumab.

The NAB2-STAT6 gene rearrangement, a hallmark of SFTs/HPCs, leads to overexpression of *EGR-1*, a potent transcriptional activator. Target genes of *EGR-1* include receptor tyrosine kinases, such as platelet derived growth factor receptor (PDGFR) and vascular endothelial growth factor receptor (VEGFR) [11,17,42,74]. Consequently, tyrosine kinase inhibitors (TKI), such as sunitinib [74], pazopanib [60] and axitinib [71], have been used to treat patients with SFTs/HPCs with good results [71,74,75,76].

Stacchiotti et al. evaluated the response to sunitinib in ten patients with metastatic SFTs [74]. Six patients showed partial response, two patients had stable disease, and three had progressive disease according to Choi criteria. Pazopanib activity was recently evaluated in a prospective, multicenter, single-arm, phase 2 trial [76]. Thirty-one patients were included in the response analysis with 18 patients (58%) showing partial response, 12 patients (39%) had stable disease and one patient (3%) had progressive disease according to Choi criteria. Median progression free survival was 9.8 months, and the overall survival was 49.8 months. Six patients (19%) discontinued treatment due to toxicity. In 2019, an exploratory, investigator driven phase 2 clinical trial explored the activity of axitinib in 17 patients with metastatic SFTs [71]. All patients had progressive disease within 6 months of entering the study according to Choi criteria. The best Choi response was a partial response in seven (41.2%), stable disease in six (35.3%) and progressive disease in four (23.5%) patients. The median progression-free survival was 5.1 months, and the median overall survival was 25.3 months.

In our case series, one patient (#2) received systemic therapy with axitinib while another (#3) received pazopanib. Patient #2 showed no response and developed progressive disease. Patient #3 developed complications associated with systemic pazopanib therapy and the treatment had to be discontinued.

As mentioned above, hypoglycemia is commonly associated with SFTs/HPCs due to paraneoplastic IGF secretion. Interestingly, *IGF2* is a target gene of *EGR-1* and binds to IGF1R, a receptor tyrosine kinase, which leads to cell proliferation through the PI3K/Akt/mTOR signaling pathway [77]. Hence, blocking the IGF/IGF1R pathway may be a promising target in treating SFTs/HPCs [74,77].

Liver transplantation (LT) has been reported as a treatment option for patients with metastatic SFT/HPC disease to the liver [34,61,64]. In two cases, the patient had severe life threatening recurrent hypoglycemic episodes, which subsided following an LT. In the third patient, resection of the hepatic metastases was deemed too risky as the patient had concurrent hepatitis B associated liver cirrhosis and a living donor liver transplantation (LDLT) was performed. However, in all three cases, the tumor recurred, two and four years, respectively, following an LT. One patient developed osseous metastases, which were resected and treated with radiotherapy. The patient has since been free of disease five years following the LT [61]. We offered an LT to patient #2 following multiple local recurrences and extensive surgical resections. However, the patient opted against an LT and started systemic therapy with axitinib, but ultimately developed a new intracranial lesion and multiple new liver lesions. Taking into consideration the short period between intracranial recurrence after an LT was recommended, the decision to offer an LT to this patient needs to be scrutinized. In summary, an LT should only be offered to highly selected patients in whom all other treatment options have been exhausted.

Strengths of this study include the multidisciplinary treatment approach as well as the relatively long follow-up period. However, our study does have limitations, most of which are inherent to the case series study design. Therefore, our findings should be considered for hypothesis generating only. Importantly, while we were able to achieve good long-term results using an aggressive treatment approach, the net survival benefit compared to a more conservative treatment strategy remains unclear. Future randomized controlled or case-controlled trials would be desirable. However, due to the rarity of the disease, conducting controlled studies does not seem to be feasible in this context. For now, results from case series seem to be the best available evidence.

## 5. Conclusions

SFTs/HPCs are rare intracranial tumors displaying various forms of malignant potential based on their primary tumor grading. Metastases to the liver may occur years or even decades after the initial diagnosis. So far, only 37 cases have been published on this topic. Once metastatic spread has occurred, only palliative treatment is possible. However, using a multidisciplinary approach long-term survival is achievable for these patients. Herein, we report three cases of patients with liver metastases from intracranial SFTs/HPCs including the longest survival observed to date (89 months following diagnosis of liver metastases), adding to the list of previously published cases (Table 5). Therefore, referral to a tertiary academic cancer center where optimal treatment can be provided is highly recommended.

## Figures and Tables

**Table 1:** WHO grading of SFTs/HPCs.

Histologic Features	WHO Grade	Old Definition
High collagen content, low cellularity	I	SFT
Low collagen content, higher cellularity, staghorn vasculature	II	HPC
Anaplastic appearance, ≥5 mitosis per 10 HPFs	III	Anaplastic HPC

HPC, Hemangiopericytoma; HPFs, high power fields; SFT, Solitary fibrous tumor; WHO, World Health Organization; Modified from Louis et al. [7].

**Table 2:** Patient Characteristics.

Case Number	#1	#2	#3
Patient age at diagnosis (years)	67	39	65
Patient sex	Female	Female	Female
Comorbidities/Notes	Parkinson’s disease	Childbed	Depression
WHO grade	II	II	II
Mitosis/10 HPF	0	2	2
Ki-67 index	5%	10–15%	2%
Liver metastasis			
Time to occurrence from initial diagnosis (years)	8	8	15
Location at initial diagnosis	Right lobe	Bilobar	Bilobar
Number at initial diagnosis	1	3	3
Size of largest metastasis at initial diagnosis	13 cm	12 cm	13 cm
Paraneoplastic syndromes	Hypercalcemia	Hypoglycemia	None
Immunohistochemistry			
CD34	N/A	Positive	Negative
CD99	N/A	Positive	Positive
STAT6	Positive	N/A	N/A
Vimentin	N/A	N/A	Positive

HPF, high powered field; NA, not available; WHO, World Health Organization.

**Table 3:** Timetable showing multimodality treatment of liver metastasis.

**Case #1**	**Treatment**	**Comments**	**Liver Segments**	**Classification**	**Complications**
05.11.2018	SRFA	1 lesion, 13 cm, 6 needles, 47 min ablation time	V, VII, VIII	Initial	none
29.11.2018	SRFA	1 lesion, 13 cm, 28 needles, 223 min ablation time	VIII	Initial	none
11.12.2018	SRFA	1 lesion, 13 cm, 13 needles, 60 min ablation time	V, VII, VIII	Initial	none
28.01.2019	SRFA	1 lesion, 13 cm, 15 needles, 43 min ablation time	V, VII, VIII	Initial	none
01.03.2019	SRFA	1 lesion, 13 cm, 5 needles, 17 min ablation time	VIII	Local recurrence	none
05.11.2020	SRFA	1 lesion, 13 cm, 12 needles, 59 min ablation time	VIII, V	Distant recurrence	none
**Case #2**	**Treatment**	**Comments**	**Liver Segments**	**Classification**	**Complications**
16.06.2015	TAE	Embozene™ Microspheres (500 μm), Soft Embolization Coils (Boston Scientific), Pushable Fibered and Detachable Non-Fibered Microcoils (Cook Medical)	VII, VIII, II, III	Initial	none
31.07.2015	TAE	Embozene™ Microspheres (2 × 100 μm, 4 × 250 μm, 1 × 750 μm)	VII, VIII	Initial	Infection of necrotic liver tissue (CD II)
22.09.2015	TAE	Embozene™ Microspheres (4 × 100 μm, 2 × 250 μm, 2 × 400 μm)	V, VIII	Initial	Infection of necrotic liver tissue (CD II)
01.12.2015	Surgery	Right hemihepatectomy	V-VIII	Local recurrence	none
15.02.2016	SRFA + liver packing	3 lesions, 3 cm max. diameter, 6 needles, 17 min ablation time	II	New lesions	none
10.11.2016	Surgery	2 lesions, Resection S II + III, 6 h 47 min	II, III	Local recurrence	none
16.03.2017	SRFA	1 lesion, 2 cm max. diameter, 3 needles, 6 min ablation time	IVa	New lesion	none
11.08.2017	SRFA	3 lesions, 0.8 cm max. diameter, 5 needles, 12 min ablation time	IVa	New lesions	none
01.02.2018	SRFA	2 lesions, 1 cm max. diameter, 2 needles, 8 min ablation time	IVb	New lesions	none
11.19–01.20	Systemic therapy	Axitinib		New lesions, local recurrence	Progressive disease under therapy axitinib (new intracranial tumor 01/20)
09.06.2020	Systemic therapy	Temozolomide, bevacizumab		New lesions	Progressive disease under therapy
**Case #3**	**Treatment**	**Comments**	**Liver Segments**	**Classification**	**Complications**
05.05.2014	TAE	Contour™ PVA Embolization Particles (255–500 μm), Pushable Fibered Microcoils (Cook Medical), Soft Embolization Coils (Boston Scientific), MicroPlex^®^ Coil System (MicroVention, Inc.)	IVa, IVb, V, VI, VII	Initial	Postembolization syndrome (CD I)
24.06.2014	TAE	Contour™ PVA Embolization Particles (150–500 μm)	II, III	Initial	Postembolization syndrome (CD I)
22.07.2014	TAE	Contour™ PVA Embolization Particles (250–350 μm)	IVa, IVb, V, VI, VII	Initial	none
10.11.2014	TAE	Embozene™ Microspheres (2 × 100 μm)	IVa, IVb, V, VI, VII	Initial	none
08.04.2015	TAE	Embozene™ Microspheres (1 × 100 μm, 1 × 250 μm, 1 × 400 μm, 1 × 500 μm)	II, III	Initial	none
06.05.2015	TAE	Embozene™ Microspheres (1 × 100 μm, 1 × 200 μm)	IVa, IVb, V, VI, VII	Initial	none
26.08.2015	Surgery	Resection of left lateral segments	II + III	Initial	Bilioma (CD I)
08.07.2016	TAE	Embozene™ Microspheres (2 × 100 μm, 1 × 250 μm)	IVa, IVb, V, VI, VII	Local recurrence	none
21.11.2016	SRFA	3 lesions, 5.1 cm max. diameter, 8 needles, 63 min ablation time	IVa, VII, VIII	Local recurrence	none
07.12.2016	SRFA	2 lesions, 2.3 cm max. diameter, 4 needles, 59 min ablation time	IVa, VIII;	Local recurrence, new lesion	none
26.01.2017	SRFA	2 lesions, 8 cm max. diameter, 9 needles, 74 min ablation time	VI	Local recurrence	Right pleural effusion, pigtail catheter (CD IIIa)
23.03.2017	SRFA	2 lesions, 8 cm max. diameter, 20 needles, 104 min ablation time	V, VI	Local recurrence	none
30.10.2017	SRFA	8 lesions, 2 cm max. diameter, 9 needles, 32 min ablation time	V, VI	Local recurrence, new lesion	none
04.04.2018	SRFA	4 lesions, 2 cm max. diameter, 10 needles, 36 min ablation time	V, VI, VII, VIII	Local recurrence, new lesion	none
05.19–08.19	Systemic therapy	Pazopanib		Distant recurrence	Leukocytopenia, Infection, Sepsis CTCAE III

CD, Clavien–Dindo; SRFA, stereotactic radiofrequency ablation; TAE, transarterial embolization.

**Table 4:** Patient Outcomes.

Case	#1	#2	#3
Treatment			
Surgery	No	Yes	Yes
TAE	No	Yes	Yes
SRFA	Yes	Yes	Yes
Systemic therapy	No	Yes	Yes
Highest CD complication	0	II	IIIa
CCI (points)	0	29.6	30.2
Adverse events			Sepsis
Patient status	AWD	AWD	DOD
Follow-up (months)	39	73	89
Time to recurrence	19 ½ months	3 ½ months	9 ½ months

AWD, alive with disease; CD, Clavien–Dindo; CCI, comprehensive complication index; SRFA, stereotactic radiofrequency ablation; TAE, transarterial embolization; DOD, died of disease.

**Table 5:** Summary of patients with intracranial SFTs/HPCs and liver metastases.

Number	Year of Publication	Author	Age ^#^/Sex	Time to Metastasis (Years)	Other Sites of Metastatic Disease	Treatment (for Liver Metastases)	Outcome (Follow-Up in Months) *
1	1959	Meredith [44]	44/F	13	Bone, Kidney, Pancreas, Mediastinum, Lung	None	DOD (N/A)
2	1960	Hukill and Lowman [45]	43/F	11	None	Surgical resection	DOD (2)
3	1971	Petito and Porro [46]	76/F	11	None	N/A (autopsy finding)	Died due to duodenal perforation following brain surgery
4	1976	Jestico et al. [47]	47/M	6	Kidney, Pancreas, Lymph nodes	N/A	N/A
5	1984	Itoh et al. [48]	49/M	6	Bone	N/A	N/A
6	1988	Yoshida et al. [49]	33/M	6	None	Surgical resection	AWD (31)
7	1991	Chakravarty et al. [50]	41/F	6	None	Surgical resection	NED (7)
8	1993	Kaneko et al. [51]	54/M	19	None	Surgical resection	NED (9)
9	1996	Sohda et al. [31]	64/F	20	None	TAE	Died due to massive liver hemorrhage following TACE
10	1998	Niwa et al. [52]	55/M	13	Lung, Bone, Pancreas	Surgical resection, Radiotherapy	AWD (84)
11	1999	Grunenberger et al. [38]	42/F	12	Lung, Bone	Chemotherapy, TACE, Surgical resection	NED (31)
12	2001	Someya et al. [53]	56/F	14	Bone, Lung	None	DOD (6)
13	2004	Spatola and Privitera [54]	48/F	10	Kidney, Breast	Surgical resection, Chemotherapy	DOD (27)
14	2004	Kim et al. [55]	62/M	7	Bone, Lung	None	AWD (24)
15	2004	Chang et al. [56]	48/F	5	Bone, Lung, Kidney	N/A	N/A
16	2004	Miyamoto et al. [57]	61/F	N/A	Bone	N/A	N/A
17	2009	Iwamuro et al. [58]	54/F	10	Bone	RFA, TACE	NED (60)
18	2010	Chan et al. [59]	49/F	7	Lung	Chemotherapy	N/A
19	2012	Chan et al. [28]	50/M	7	Kidney, Bone, Abdominal cavity,	Chemotherapy	AWD (N/A)
20	2014	Lee et al. [60]	60/M	8	Pancreas, Lung, Bone	Surgical resection, RFA, Chemotherapy	AWD (~24)
21	2015	De Martin et al. [61]	48/F	4	Bone	Liver transplant	NED (60)
22	2015	Nickerson et al. [62]	65/M	25	Abdominal cavity, Bone	Chemotherapy	AWD (~36)
23	2015	Manatakis et al. [63]	35/M	12	Bone	Surgical resection, RFA, TAE	AWD (12)
24	2015	Urata et al. [64]	58/M	16	Bone, Lung, Soft tissue	TACE, LDLT	DOD (65)
25	2016	Han et al. [65]	42/F	18	None	N/A	AWD (N/A)
26	2016	Han et al. [65]	36/M	6	Lung, Bone, Spinal cord	N/A	DOD (N/A)
27	2016	Han et al. [65]	53/F	10	Breast, Lung	N/A	AWD (N/A)
28	2016	Lo et al. [66]	35/M	5	N/A	Chemotherapy	AWD (34)
29	2016	Lo et al. [66]	51/F	19	N/A	Surgical resection	NED (26)
30	2016	Lo et al. [66]	60/M	16	N/A	Surgical resection	NED (39)
31	2016	Lo et al. [66]	31/F	7	N/A	Surgical resection, RFA	AWD (36)
32	2016	Lo et al. [66]	48/M	12	N/A	RFA	DOD (36)
33	2018	Patro et al. [67]	53/F	N/A	Bone	Chemotherapy	N/A
34	2019	Reddy et al. [17]	74/M	0	None	SBRT, Chemotherapy	AWD (18)
35	2020	Hasimu et al. [18]	62/F	6	None	Surgical resection	NED (6)
36	2020	Maeda et al. [68]	52/F	13	Lung	Surgical resection, Systemic therapy	DOD (59)
37	2021	Maeda et al. [30]	49/M	10	Lung, Kidney, Adrenal gland, Abdominal Cavity	N/A	DOD (0)
38	2022	Krendl et al.	67/F	8	Abdominal cavity	SRFA	NED (39)
39	2022	Krendl et al.	39/F	8	None	TAE, Surgical resection, SRFA	AWD (73)
40	2022	Krendl et al.	65/F	15	Bone, Abdominal cavity	TAE, Surgical resection, SRFA	DOD (89)

^#^ Age recorded at diagnosis of liver metastases; * Duration from diagnosis of hepatic metastasis to last follow-up or death; AWD, alive with disease; DOD, died of disease; LDLT, living donor liver transplantation; N/A, not available; NED, no evidence of disease; RFA, radiofrequency ablation; SBRT, stereotactic body radiation therapy; SRFA, stereotactic radiofrequency ablation; TACE, transarterial chemoembolization; TAE, transarterial mechanical embolization.

## Data Availability

All data used are available but unsuitable to publish or post because patients participating in this case series were assured raw data would remain confidential.
